# A Longitudinal Observational Mixed Methods Cohort Study of the Psychosocial and Financial Costs of Cancer Care for Pediatric Families in the Maritimes, Canada

**DOI:** 10.1177/10732748261468826

**Published:** 2026-09-11

**Authors:** Zara Nicole Forbrigger, Derek Bruce MacDonald, Tamara MacDonald

**Affiliations:** 1 Department of Hematology and Oncology, 3682 IWK Health Centre, Halifax, NS, Canada; 2 Department of Pharmacology, Dalhousie University, Halifax, NS, Canada

**Keywords:** pediatric oncology, financial burden, psychosocial, employment, cost of illness

## Abstract

**Introduction:**

Approximately 1,000 children are diagnosed with cancer in Canada each year. Treatment places significant financial and psychosocial burdens on families through direct medical costs and indirect costs such as travel and lost income. These burdens can negatively affect parental quality of life. In the Maritimes (Nova Scotia, New Brunswick, and Prince Edward Island), all pediatric cancer care is centralized in Nova Scotia, requiring many families to travel long distances for treatment. However, the impact of this travel burden on Maritime families has not yet been studied.

**Methods:**

A prospective, longitudinal, observational mixed-methods cohort design was used for this study. This study examined the indirect and psychosocial costs of families treated at the IWK Health Centre through capturing expenses and interviews respectively. The study period was 2016-2020 and included families who had a child being treated for cancer. Thirty families participated in the study, with 28 providing financial information and 29 participating in interviews. Financial information was collected during the first year of treatment. Semi-structured interviews were used to capture the psychosocial impact. Interviews were transcribed and thematically analyzed using NVivo by two independent researchers.

**Results:**

Families spent a median of $9668 (IQR $7944-12958) on indirect costs and had a median of 55 (IQR 0-125) days of income lost. Most families (79%) in our study experienced a psychosocial burden using terms like stress, worry, or frustration directly as a result of the financial burdens from cancer treatment, in addition to the already significant anxiety around having a child with cancer.

**Conclusion:**

The financial hardships of cancer treatment have lasting negative implications on families. Current programs and infrastructure are not sufficient to support most families in Canada. There is a need to expand caregiver support programs so families can focus on caring for their child with cancer without suffering financial toxicity.

## Introduction

Around 1000 children are diagnosed with cancer every year in Canada.^1^ Treatment of cancer results in three main costs to families: direct, indirect, and psychosocial. Direct costs are out-of-pocket health care expenses that include medications and medical equipment.^2^ Indirect costs are any expenses due to their diagnosis but not directly related to treatment such as lost wages, household upkeep, or travel related expenses.^3^ Psychosocial costs affect the quality of life of families and patients and include the anxiety and depression related to having a child diagnosed with cancer.^4^ Studies by Tsimicalis et al. in 2012^5^ and Oliveira et al. in 2018^6^ examined the health care costs of cancer in Canada and determined that they were steadily rising. The overall costs of illness for pediatric patients in the Maritimes have not been assessed and studies exploring these costs for this population are sparse.

In Canada, there are 16 pediatric hospitals, one of which is the IWK Health Centre (IWK). The IWK is a tertiary hospital located in Nova Scotia (NS), which has a shared-care model with two other Canadian provinces, New Brunswick (NB) and Prince Edward Island (PEI). These three provinces make up the Maritime Provinces. The shared-care model is a collaboration between the IWK and health professionals at the patients’ home communities to make it possible for children to receive care close to their home as frequently as possible. However, families diagnosed with cancer anywhere in the Maritime Provinces must still travel to the IWK in NS for some treatment. The shared-care model distributes lower levels of treatment to local hospitals, thereby reducing the need for families to travel to the IWK for all treatment, families experience long travel times and increased costs overall.

There are many direct and indirect costs associated with pediatric cancer treatment. Most direct costs in treatment are covered for all patients while in hospital. However, indirect costs such as travel, lodging and meals are not covered for families attending treatment and incurring these expenses. In many cases, families receive help at the hospital with meals, parking, pre-paid vouchers to take taxi rides at a reduced cost, but there are no formal programs to guarantee all families have consistent help throughout treatment with any of these indirect costs. In addition, the psychosocial costs associated with treatment often increase with increasing indirect costs, particularly in families with limited financial resources.^7^ Therefore, this study focused on the indirect and psychosocial costs of treatment, investigated quantitatively and qualitatively respectfully, which as the reader will see, represent a significant and powerful burden on these families.

This is the first study to determine the financial impact on families and the associated emotional burden while caring for a child with cancer in the Maritimes, Canada. Our research question was, “What are the financial costs and psychosocial burdens from caring for a child with cancer?” The aim of this study was to determine what indirect expenses families experience due to travelling for treatment and how these affect families’ psychosocial well-being. Understanding these impacts is necessary to inform policy and increase support for families.

## Methods

A prospective, longitudinal, observational concurrent mixed method cohort design was utilized. Phenomenology and content analysis methodological orientation was used. Ethics approval was obtained from the IWK Health Research Ethics Board (#1013449) in March of 2013. Multiple instruments, methods and models were utilized to choose and collect demographic, financial and psychosocial data. The reporting of this study conforms to the STROBE guidelines.^8^

A prospective convenience sample model was used to recruit all participants who were parents (or the person financially responsible) whose child was: aged 0-18 years; spoke English; diagnosed with any cancer in the previous 6 months; receiving treatment at the IWK between 2016 and 2020; and having been offered curative therapy. Participants were approached face-to-face. To minimize selection bias, all families meeting this eligibility criteria were approached for participation at clinical visits when study personnel were available. All participants were informed during consent that this was a mixed-methods model utilizing multiple data collection strategies and gave written consent. A mixed-methods design was chosen as combining financial records with interviews provides a comprehensive understanding of the impact of travelling due to cancer treatment on families. Quantitative financial data objectively measures the economic burden, including indirect costs, lost income, and out-of-pocket expenses. Qualitative interviews complement these findings by exploring the emotional and psychological experiences associated with financial stress, such as anxiety, uncertainty, guilt, coping challenges, and effects on family relationships. Integrating both methods allow researchers to connect measurable financial hardship with lived experiences, improving the depth, validity, and contextual interpretation of the findings. This approach also helps identify issues that may not be captured through financial data alone, providing a more patient- and family-centered understanding of burden and informing supportive interventions and policy development. All patient details have been de-identified.

The Family Information Measure (FIM)^9^ was used to collect demographic, distance to hospital, and family income data. The questionnaire was given to the families at the time of enrolment. The research assistant helped each family complete the questionnaire during the initial interview. The Ambulatory and Home Care Record (AHCR) was used to collect quantitative costs data. The AHCR was developed as a comprehensive and adaptable instrument to value health resource costs associated with ambulatory and home-based care from the perspective of the families incurring those costs.^10^ Quantitative data was reported monthly for the first year of treatment using diaries, receipts, medical records, and interview data. Participants provided written records of expenses or receipts they collected where possible for a direct calculation of costs incurred. Otherwise, the research assistant recorded verbal reports of costs and/or estimated the costs using a standardized method.

### Meals

To calculate the total number of meals during an IWK Health treatment visit, participants medical records were accessed to record their visit dates, duration of stay, and the number of family members present during the visit. We adopted the following standardized method to estimate the cost. We estimated that the average individual eats 3 meals a day (breakfast, lunch, and dinner) with 3-4 hours between each meal. At the IWK Dial for Dining is a meal service program that delivers food to the patient’s room with the cost being covered for the patient by health care. Dial for Dining, at the time the study took place, had a meal cost breakdown of $21-$27/day. We chose to use the maximum of $27/day/person, as families do not always use Dial for Dining and may resort to other more expensive options (ex: ordering out). For inpatient visits, the child’s meal is provided, thus we multiplied $27 by the number of family members present excluding the child. For outpatient visits, families were not necessarily present for all meals. Therefore, both the duration of time spent at the IWK, and travel time were used to estimate the number of meals consumed. Because meals are not provided for the child during outpatient visits, a cost of nine dollars per person per day (derived from $27 divided by three meals) was applied and multiplied by the number of individuals present, including the child.

Outpatient meals:(time at visit+time driving) * (number of meals) * (family members+child)

Inpatient meals:(time at visit) * (number of meals) * (family members)

### Mileage

Both gas expenses as well as wear and tear on a vehicle under Mileage were captured. These were calculated using mileage rates by year and province. Participants self-reported the distance (km) from their house to the hospital.

Mileage:(distance from house to IWK*2) * (number of trips to hospital) *(mileage rate)

### Parking

Rates were calculated using the standard IWK parking rates multiplied by the duration the participant was at the IWK.

Parking costs:(time at visit) * (parking rate)

### Bridge (Halifax and Confederation) and Highway Tolls

Tolls and bridges were calculated based off the number of visits to the IWK and the participants location. Halifax bridges were $2 for each trip ($1 each way) and the Cobequid Pass was $8 for each trip ($4 each way). The Confederation Bridge only has a toll one-way, so the fee was added to each visit based off the year.

### Time Lost at Work

Time lost from work was captured through participant interviews and the Family Information Measure. For individuals receiving Employment Insurance (EI) benefits, which replace up to 55% of income, the number of days off work per month was multiplied by 0.45 to estimate lost earnings. For those receiving long-term disability benefits, which typically replace 60–70% of income, days off work were multiplied by 0.35. These calculations provided an estimate of income loss expressed in equivalent workdays for each family. However, because EI benefits and long-term disability programs have maximum benefit caps, the true income loss may be underestimated in families whose earnings exceeded these thresholds. For participants not receiving EI benefits, long-term disability, or other income replacement supports, each missed workday was counted as a full day of lost income. Weekends and statutory holidays in Canada were excluded from all calculations of missed workdays.

For qualitative data collection, scheduling and execution of interviews was based on participants scheduled visits to the IWK for treatment appointments and were primarily face-to-face, all with the same interviewer. Some telephone interviews were conducted if family designates were unable to meet during appointments. The interviewer was a female research assistant with a B.Sc. trained by the study investigator with a Pharm.D and the qualitative methodologist. There was no relationship with participants prior to study commencement. Participants were informed that the research study was being conducted by researchers interested in improving the financial supports for families undergoing cancer treatment at the Maritimes, Canada. Some telephone interviews were conducted if family designates were unable to meet during appointments. Qualitative information relating to costs incurred and emotional burdens were captured through semi-structured open-ended interviews.^11^ Semi-structured interviews were conducted with families, only if they were currently on treatment or had recently finished. We asked four open ended questions in the interview: Tell me about your spending so far (probes for fuel, accommodations, food, childcare, parking and tolls); Has your employment status changed?; How do you feel about the amount of money you are spending due to travel because of treatment?; Has this impacted you financially in other areas? The duration of interviews ranged from a few minutes to half an hour depending on the amount of information the participant disclosed. Transcripts were not returned to participants to comment or correct the data.

## Statistical Analysis

Descriptive analysis was run on data collected using the Family Information Measure. Significance was determined using a paired t-test when comparing changes in cost within a patient between the first and last 6 months of the first year of treatment. Six months was chosen based on children completing therapy and transitioning to outpatient treatment at this time. Independent samples t-tests were used to compare in province vs out-of-province expenses. Pearson correlations were used to determine if there was a correlation between total expenses and distance to the IWK or number of inpatient visits. Missing information was coded using a placeholder value (999) and treated as missing during analysis. Variable-specific analyses were conducted using available cases only. A p-value of <0.05 was used for significance. With a large effect size, a minimum of 28 participants is required to reach statistical power of 0.8 with an alpha = 0.05.

All audio recordings of qualitative data from face-to-face and telephone interviews were transcribed verbatim. The transcription was imported into QSR NVivo 2.0 qualitative research software. Data was analyzed based on Strauss and Corbin’s (1990) technique of Constant Comparative,^12^ in which the data from all interviews and participants is read and re-read, coded and re-coded until consistent and coherent themes emerge to the qualitative data analysis team. The data was reviewed by two independent researchers and themes were generated separately and then discussed. The persistent codes and code groups became themes for the basis of the qualitative results.

## Results

### Demographics of Participants

Forty-eight families were approached for enrolment in this study. Thirty-nine agreed to participate and signed a consent form, of which nine withdrew or were lost to follow-up. A total of 30 families participated in this study through providing financial information and/or participating in interviews. Twenty-eight families provided financial information, of this 28% (n = 8) provided records of expenses and the other 78% estimated. Twenty-nine families participated in interviews, with 10 families interviewing twice to capture the full first year of treatment. Twenty-nine (74%) of the interviews were conducted in person, with the remainder occurring over the phone.

The male-to-female ratio for the child was 0.67:1. The main cancer diagnosis included in our study was leukemia’s (37%), followed by other cancers (27%), sarcomas (20%), and lymphoma (17%). Other cancers consisted of patients with Wilms tumor, brain tumors, neuroblastoma, and Langerhans cell histiocytosis. The mean age of the child with cancer was 8.32 ± 5.23 years and the mean age of the parents was 40.56 ± 7.80 years. There was an average of 4 ± 1 family members living inside the home. Most families (77%) included in this study lived greater than 100 km away from IWK Health with a mean distance of 278.73±178.15 km. It took families an average of 2:55±1:54 hours to get to the IWK not during rush hour and 3:27±1:57 hours during rush hour. Fifty three percent of patients were from NS and 47% were out of province, either NB or PEI. (Table 1). The average annual household amount was $80,000.

**Table 1.** Demographic Data on Participants

​	N = 30
Sex N(%)	
Male	12 (40)
Female	18 (60)
Diagnosis	
Leukemia	11 (37)
Lymphoma	5 (17)
Other	8 (27)
Mean Age Child (SD)	8.32 (5.23)
Mean Age Parent Age (SD)	40.56 (7.80)
Number of family members	4.03 (0.96)
≥100km away from hospital N(%)	23 (77)
Mean Distance to hospital	278.73 (178.15)
Mean Time to hospital not during rush hour	2:55 (1:54)
Mean Time to hospital during rush hour	3:27 (1:57)
Province N(%)	
Nova Scotia	16 (53)
Out of Province	14 (47)

### Quantitative Analysis

#### Travel Costs (Mileage, Food and Accommodations)

There are many expenses associated with pediatric cancer treatment. This category of travel costs represents both the most varied and the most impactful category. As expected, as distance to the hospital increased so did travel expenses (r(26) = 0.70, p < 0.001). However, families ranging from local to out-of-province experienced increase costs relating to travel and the burden that it caused. Families spent a median of 50.5 days (IQR 23-85) as inpatients. The duration of inpatient stays within the first year was weakly correlated to the total cost but was not statistically significant (r(22) = 0.39, p = 0.059). Families described costs relating to fuel, accommodations, food, tolls, and parking. In this study the average amount of money spent on mileage was $4620 (IQR 3545-6568) in the first year of treatment (Table 2).

**Table 2.** Total Costs Incurred by Participants Over the First Year of Treatment for Childhood Cancer

Expense	Median (IQR), CAD
Accommodations	136 (0-1220)
Food	2765 (2459-3476)
Mileage	4620 (3545-6568)
Other	0 (0-303)
Parking/Tolls	660 (463-858)
Total^*^	9668 (7944-12958)

*Medications were not included in the total expenses for patients.

One family described this expense as:

*“Fuel is the biggest, the absolute biggest. To get here we’re looking at about $600-800 month, that’s just gas to get here, so that he can have treatment.”*

**Figure 1.**
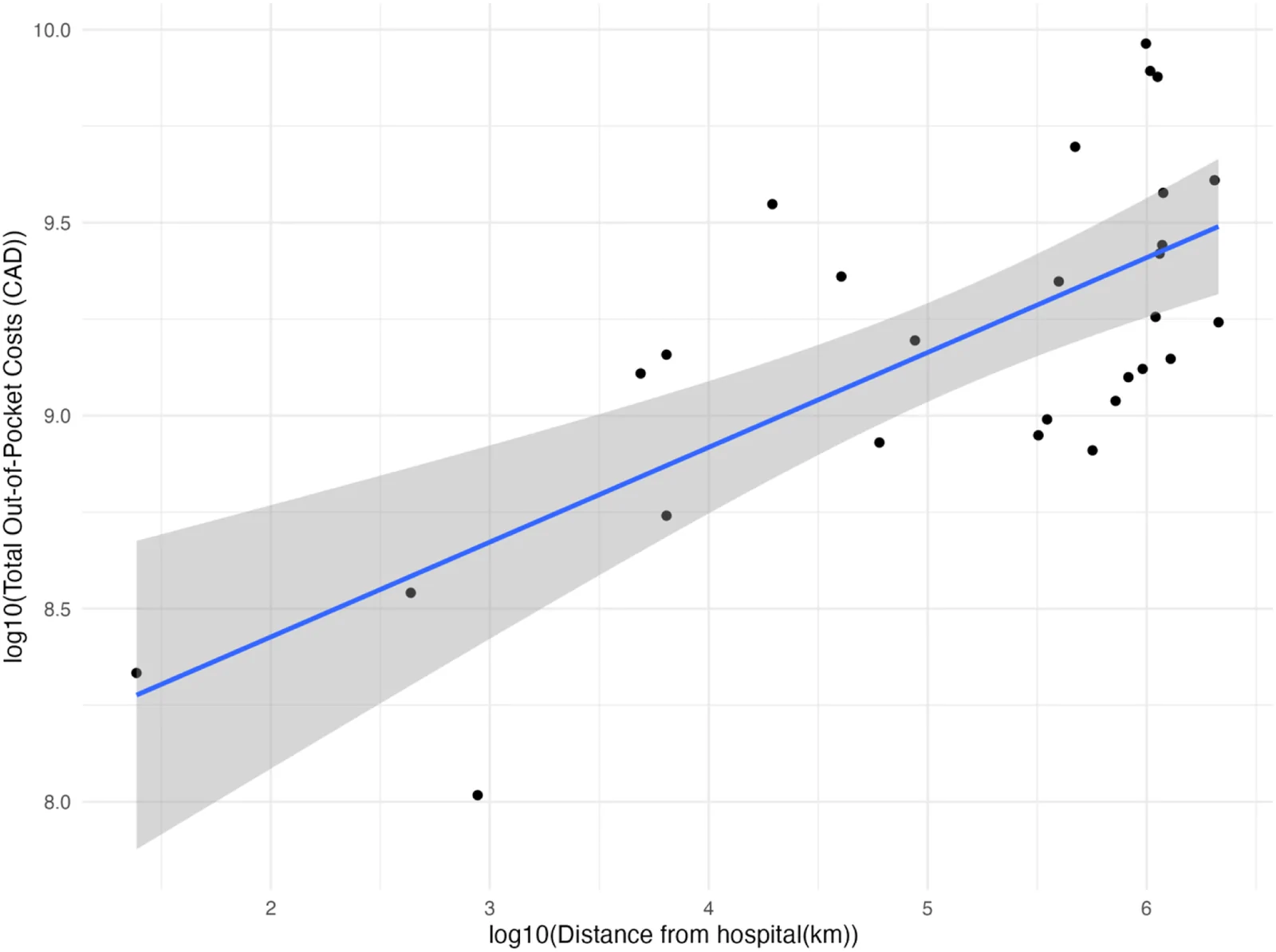
Pearson correlation between the log_10_ transformed distance from the participants house to the IWK (km) and the log_10_ transformed total indirect money (CAD) spent on travelling for treatment with 95% confidence interval. Pearson correlation, r(26) = 0.70, p < 0.001

As a result of the Maritimes being vast geographically, we assessed the impact of distance from the tertiary care hospital. Pearson correlation showed that distance to the hospital and total money spent was correlated (p=<0.001, Figure 1).

In our study we estimated that families spent a median of $2765 (IQR $2459-3476) in the first year of treatment on food (Table 2), with most expenses occurring in the first 6 months (p<0.001, Table 3). One family described food as being the largest cost incurred:

*“The majority of our money was spent with food…when you have a child that’s in treatment it’s really difficult to stimulate an appetite to begin with so it was very quick that the food from the cafeteria became something that she just couldn’t bring herself to eat”*

**Table 3.** Costs Incurred by Participants During the First Six Months of Treatment Compared to Months 7-12

Expenses	1-6 months	7-12 months	P value
	Median (IQR)	Median (IQR)	
Accommodations	125 (0-815)	0 (0-229)	0.010
Food	2249 (1906-2524)	558 (402-1016)	<0.001
Mileage	3313 (2274-4982)	1472 (640-1788)	<0.001
Other	0 (0-119)	0 (0-182)	0.626
Parking/Tolls	470 (373-589)	168 (67-273)	<0.001
Total^*^	3541 (2641)	1184 (688-1710)	<0.001

*Medications were not included in the total costs.

This quotation highlights an additional concern for families. As a result of cancer treatment, children may experience altered taste and reduced appetite. When a child is unable to consume meals from the cafeteria or Dial for Dining services, families are required to purchase meals out-of-pocket rather than having them covered, thereby increasing overall expenses. This suggests that the meal costs estimated in this study may underestimate the true financial burden incurred by families.

Depending on the family, the biggest financial concern differed. This highlights how each families experience is unique and multiple areas of spending should be addressed to support all families.

Sixteen (55%) families reported having to spend money on accommodations. Families reported spending money on the Ronald McDonald house or hotels. The estimated total costs spent on accommodations in the first year was $136 (IQR $0-1220) (Table 2). The families that did not have costs associated with accommodations either lived close to the hospital, were admitted to the hospital, or had family/friends that would provide lodging. In total families spent an average of $9668 (IQR $7944-12958) indirect (travel) costs in the first year of treatment (Table 2). The differences in costs for families did not vary per province (Table 4). This may be explained by the geographic distribution of families in Nova Scotia. Families living in the northern region, may need to travel 400 km to reach the IWK. In contrast, patients close to the border of Nova Scotia for example in Moncton, New Brunswick, would need to travel 265 km to the IWK. These findings highlight that the burden is not strictly province-dependent, supporting the finding that distance is the important factor when considering expenses.

**Table 4.** Costs Incurred by Participants Who Live in Nova Scotia or Outside of Nova Scotia (Prince Edward Island or New Brunswick)

Expenses	In nova scotia	Out of province	P value
	Median (IQR)	Median (IQR)	
Accommodations	0 (0-196)	671 (0-1282)	0.952
Food	2799 (2444-3260)	2686 (2583-3780)	0.346
Mileage	4200 (2929-6827)	5551 (4000-6260)	0.472
Other	0 (0-250)	0 (0-500)	0.659
Parking/Tolls	819 (471-906)	581 (448-750)	0.198
Total^*^	9490 (6905-11974)	10320 (8950-14430)	0.384

*Medications were not included in total expenses.

### Qualitative Analysis

#### Finding: Non-Institutional Help Is Unpredictable

Most families (22/29, 76%) reported receiving some form of help, which came in many different forms. The IWK Social Workers were directly mentioned by 11/29 (38%) families as being helpful for providing meal, parking, and gas cards. Fifteen (52%) families mentioned receiving help from their families and friends which included travel to stay with the family during treatment, taking care of siblings, offering their homes, and offering to help financially:

*“My in-laws have helped us out financially… so we haven’t worried about our bills or our mortgage or anything.”*

Some form of “community” was another source of help mentioned by 8/29 (28%) families. This included community groups fundraising, holding auctions, as well as neighbours preparing food and taking care of family assets while away at treatment:

*“We had some fundraisers at her school and stuff… I don’t know what we would have done if we hadn’t had those… they were huge helps. Just to stay above water.”*

Despite most participants reporting receiving some form of help, there was a wide range of help or support received among the families in this study. Some families report receiving no help (6/28, 21%).

“*I don’t know how we’re ever going to get out of the hole that we’re in…There’s no help for us. There’s zero help for us.”*

One family mentioned that it is not just the lack of help but how difficult it can be to get the help they are entitled to:

*“I’m more upset at how hard you have to work to get help or how many walls you have to knock down in order to find the help or…when they say no sorry can’t help.”*

#### Finding: Recovering Costs Through Income Tax Is Disappointing

Income tax claims are one option for caregivers to recover some indirect expenses. However, it was clear that families struggled with this, not only in figuring out what expenses could be claimed, but also how to accomplish this. Seven (24%) participants mentioned using income tax to recover travel expenses. Some families described disappointment in the amount of the tax return:

*“We’ve tried to record what we’re spending for purposes of income tax and that’s kind of disappointing that we don’t get better discount or a better deduction.”*

### One Family Was Unable to claim Any Medical Expenses

“*I had all my receipts done up and it was just over 2 grand…It wasn’t enough for me to claim it on income tax as a medical benefit.”*

The overall sense was that participants felt they were not able to use their tax returns to substantially recover their indirect expenses paid.

### Finding: Healthcare Coverage Is not Universal

Although it was not specifically asked in interviews, there was a common expectation among participants that Canada’s universal healthcare system would cover all direct treatment expenses. Insurance was also expected to cover other direct treatment expenses such as thermometers, shower chairs, wigs, knee braces, and other medical supplies, all of which were specifically mentioned by interviewees. Eight (28%) participants indicated that these expenses were either only partially covered or not covered by universal healthcare or insurance. One family summed it up as:

*“That perception of universal health care, you know it doesn’t cost anything for health care here in Canada…We’re living the fact that that’s not entirely true.”*

Families also experienced other costs that were unexpected or arose earlier than anticipated. A recurring example was having to expedite vehicle repairs or purchase a new vehicle to ensure a reliable means of transportation to the hospital. These expenses accumulate over time and add to the financial burden families experience on top of direct treatment.

### Finding: Caregiver Employment Insurance Is Underwhelming

The expectation with many of the participants was that Employment Insurance Caregiving Benefits would help when they are unable to work due to a critically ill child, which was not the case. EI benefits are to a maximum of 55% of earnings for up to 35 weeks. In addition, there is an initial interval of time after you apply in which you do not receive benefits. There are also some circumstances when EI is not available, such as in the summer months for teachers or during active maternity leave. One participant shared their frustrations:

“*It’s not really fair that the only benefit you can get is if you’ve been working full time for a year before they got sick because you don’t plan for this kind of stuff right? It’s really, really frustrating. That’s the biggest emotion I have with all this.”*

Another group of people who are ineligible for EI are individuals who are self-employed:

*“Because I am self-employed, I don’t have the luxury of unemployment or sick benefits or you know mental health benefits, I don’t have that option.”*

### Finding: Lose Income or Take Care of Patient

Most participants (20/28, 71%) experienced a loss of wages. The common stages for participants who went off work to retain income was to first consume sick leave, then vacation leave, then EI benefits, and then resort to some form and schedule of unpaid leave, culminating, for a few participants, in leaving their employment or being fired. In our study there was a median of 55 (IQR 0-125) days of work missed during the first year of treatment, with a range of 0-346 days. This is an average of 3 months of lost wages meaning for a family earning $80,000 this results in a $20,000 reduction.

### Finding: Psychosocial Burden Is Overwhelming

The burdens created through the experiences of cancer treatment are financial and emotional, with the emotional burden from the diagnosis itself amplified by financial burdens. Although employment and financial circumstances may differ, 79% (n = 23) of families described a psychological impact of expenses related to travel. This included the terms “stress”, “difficulties”, “worry”, “frustration”, and “shock”. One family described it as:

*“The financial side of it has just been, it’s upsetting, it’s stressful, it’s debilitating.”*

Another indicated that:

*“I’ve got so much going on, so much on my mind, that I can’t really even remember words anymore.”*

These statements demonstrate the extreme emotional distress families experience due to the diagnosis itself coupled with the financial burden. It is important to note that although the foci of this study were the actual costs and the resulting psychosocial burdens from those costs, the presence of existing and significant anxiety from the cancer diagnosis itself reduced some families’ capacities to shoulder more burden, amplifying the effects of the psychosocial burdens due to the increased financial costs. All these current findings should be perceived in addition to this existing anxiety.

Many families faced the decision to go back to work and reduce caring for their child while maintaining an income, or to stay with the child during treatment and accept reduced income. All this time, expenses either stay consistent or in the case of most families, increase. A few families (5/29 or 17%) were “ready” for this financial burden, but most were not, facing expenses beyond their means.

### Finding: Participants Experienced Feelings of Fortune And/Or Luck

Despite the financial and emotional burdens discussed in the prior themes, many families (23/28, 82%) expressed a sense of luck and/or fortune for specific situations. The terms “fortunate” and “luck” were used 98 times across all interviews by participants while referring to their appreciation for their jobs, family, home, vehicle, health plans, accommodations, and community support. While maintaining that they were “lucky” or “fortunate” in their current situation, many families were quick to add that other families may not be as lucky:

*“We’re pretty fortunate in the sense that we both are professionals with good incomes and so we’re perhaps more able to absorb the impact of, of the financial hit than others.”*

## Discussion

There are substantial financial and psychosocial burdens associated with caring for a child undergoing cancer treatment. In this study, we enrolled 30 caregivers who resided varying distances from the tertiary care centre, ranging from a few kilometres to several hundred kilometres away, and who were required to travel for their child’s treatment. Most families reported incurring significant indirect costs, which contributed to both financial hardship and psychosocial distress. The qualitative findings reinforced the quantitative results, demonstrating a close relationship between financial and emotional burden. Families experiencing greater financial strain frequently described heightened levels of stress, anxiety, and emotional exhaustion during interviews, highlighting the interconnected nature of economic and psychosocial challenges throughout their child’s cancer treatment.

The present study found a significant financial burden for families that must travel for pediatric cancer treatment in the Maritimes, Canada. In addition, families expressed in the interviews that they had experienced a reduction in income due to changes in employment and time lost at work. Consequently, most families in our study incurred significant out of pocket costs that were supported by reports during interviews that they experienced as a direct result an emotional burden, expressing feelings of anxiety and frustration. We know that there is already a huge mental toll on families when their child is diagnosed with cancer.^4^ Financial strain is a compounding factor that effects families at a time when they are already overwhelmed with caring for a sick child.

We found that there was a significant emotional burden due to finances in most (79%) families included in this study. This finding has been reported in multiple studies where caregivers of children with cancer report an emotional impact of their financial situation.^13-18^ One study conducted in the United States used research instruments to determine caregivers’ mental state. They found that parents with higher perceived financial strain also had higher levels of depression, anxiety, and post-traumatic stress symptoms.^13^ This demonstrates the real mental health effects on caregivers due to the financial burden imposed by cancer treatment. Another study received survey responses from the US and Canada and looked at the effect on children who were aware of the financial situation. Parent’s indicated that it led to psychological outcomes in their child like guilt, anxiety, feeling like a burden, or that other family members were more deserving of the family’s resources.^19^ Our study reports that families incur significant emotional burden from the financial costs of caring for a child with cancer. The financial strain due to pediatric cancer can have a significant emotional impact on all family members.

The indirect expenses incurred by families relating to travel, on a median of $9668 (IQR $7944-12958) in the first year, was a significant burden. The families that travel longer distances sustain higher costs (p < 0.001) but most families, regardless of distance, experienced a financial burden due to cancer which may not have resulted in statistical significance due to low numbers of participants. This identifies the need to ensure travel expenses are provided for all families. Many studies have reported the costs that families experience due to treatment^5,18,20-24^ with each finding a significant amount to be attributed to their geographic location in relation to the treatment hospital. A study by Ren et al conducted in China found that 48.3% of costs incurred by families was for direct non-medical and indirect expenses relating to their child’s diagnosis.^20^ This aligns with our finding that expenses relating to non-medical costs are substantial and an area that needs to be addressed. A study conducted in Australia showed that major contributors to financial strain were found to be parking fees, gas, and other expenses related to travel,^22^ similar to our findings. At the IWK some families sometimes receive gas or meal cards. Although families indicated that it was helpful, it was not enough to eliminate the financial strain. Gift cards are provided through donations and therefore not always available for all caregivers and/or not available every visit. There is a disconnect between the financial burdens experienced and the supports provided at the provinces to remedy these burdens.

A study by Tsimicalis et al. conducted in Ontario, Canada in 2007 found that families spent $5446 CAD over a 3 month time.^5^ Unlike the present study, this included home medication costs. The real expenses caregivers experience is likely much higher than reported in the current study, especially when considering medications and inflation over the past 10 years. Another study by Nipp et al. had a unique finding when surveying survivors of childhood cancer.^23^ In pediatric oncology, ensuring patients come to the hospital for appointments is crucial to good survival outcomes. Parents being unable to afford to take their child to the hospital for treatment can result in major difficulties in appropriately caring for the child and potentially effect treatment outcomes.

Loss of employment income was a significant burden for families in the current study. Employment Insurance (EI) caregiving benefits provide up to 55% of an individual’s earnings for a maximum of 35 weeks; however, many participants felt that this support was insufficient given the prolonged duration of their child’s treatment. Families in this study reported a median loss of 55 days of paid employment during the first year following diagnosis. This reduction in income occurred at a time when household expenses were increasing due to treatment- and travel-related costs, compounding the overall financial strain. The impact was particularly pronounced among self-employed families, who are generally ineligible for EI caregiving benefits and therefore lack access to this source of income replacement. Ren et al. conducted a study in China that looked at the amount of time parents lost at work over the duration of acute lymphoblastic leukemia treatment. They found that parents missed an average of 14.4 months of work for the father and 18.1 months for the mother.^20^ This is an important consideration when looking at the results of this study, as caregivers continue to need time off even after the first year of treatment. In Ontario, Canada a study was conducted that found the total time cost for families in the first 3 months was $22873, which is a significant amount of income lost especially when expenses have increased.^5^ Multiple other studies have also determined that time lost at work had a negative impact on caregivers of a child with cancer.^14,17,22,25,26^ The current study reports a median of 2 months of lost wages in the first year, which results in a 17% lost income per year.

Hjelmstedt et al. in Sweden examined the barriers and facilitators for returning to work and found that flexibility, accommodations, and reduced childcare demands were all beneficial. They also found that access to assistance, private financial support, and continued full pay would all help meet financial needs.^26^ It is important to consider what resources are helpful for parents once they must go back to work full time. It is also important to ensure caregivers are not going into debt while they are having to take care of their child with cancer. Another study of interest found that thirty-two percent of families found that employment was less important to them now and instead wanted to focus on spending time with their families.^22^ This highlights that families with a child diagnosed with cancer choose to focus on spending time and taking care of their sick child, increasing their financial burdens as a result.

One important theme that emerged in our study was caregivers receiving help from their community and family. Previous research studies have shown that families are better able to cope with the financial strain when they have institutional and/or support networks.^17,18,22,24^ Kelada et al. conducted a study in Australia that found factors that protected against financial toxicity were loans, donations, gifts, and government payments.^22^ However, as shown in both this paper and our study, not all families have support, which can be devastating by increasing the financial and psychosocial burdens. Support for families should be equitable: whether a caregiver is able to survive financially should not be dependent on something inconsistent like donations and having family nearby. The impact is substantial emotionally and financially for families who do not receive enough help.

This study has shown that families with children in cancer care experience extra burden because there is a lack of comprehensive support in helping them address their extra costs incurred during treatment from travel, lodging, parking, meals, babysitting, missed work and missed wages, and costs not covered by existing programs. Future studies should focus on what support systems would be most beneficial to caregivers from the perspective of caregivers themselves especially programs that help reduce the cost of travel for care and lost wages. There was a burden associated with indirect costs in the year 2016-2020. The cost of living has only continued to rise in Canada and participants that spent $10000 in 2016 would be looking at spending $12735.^27,28^ It is imperative that changes are made to cover expenses that caregivers experience due to their child’s treatment, as without these interventions this problem is likely to get worse. Expanding coverage for expenses relating to travel for treatment, would help prevent this financial burden, for example travel for care within the province of the tertiary centre is not supplemented and limited to 3 consecutive day appointments for out of province families.

The caregiver support provided by the governments in the Maritimes, Canada needs to be extended for families of children with a sick child. Additionally, families who are unable to access EI benefits require a separate support system during these times as they are particularly vulnerable to financial instability. Overall, government assistance should be providing more support to families with a child with cancer to facilitate parents being caregivers for their child.

## Limitations

Limitation of this study included potential inaccuracy in parent self-reporting of finances possibly resulting in underreporting of costs. This study did not include all out of pocket costs e.g. medication costs as this was thought to be less of an impact due to government, 3^rd^ party and compassionate supply of medications in Canada. This may have led to underestimates of costs. Another limitation of the present study was that home hospital visits were not captured, reporting only occurred at the tertiary centre. Additionally, the calculation of money spent on food was an estimate. The constant recording of all food costs added an additional burden to families and chose to use a cost estimate for families who did not provide information. This estimate is based on the family eating 3 meals a day from the hospital cafeteria. Any food consumed outside of the cafeteria would result in additional costs to families that was not captured.

In addition, there may be inaccuracies in the recorded number of family members present during hospital visits, as data collection occurred at specific times of day and may not have captured all individuals accompanying the child. Consequently, estimates of food-related expenses should be interpreted as approximations rather than exact values.

The estimated loss of employment income may underestimate the true financial impact on families. Employment Insurance (EI) caregiving benefits and long-term disability programs provide income replacement only up to a maximum weekly benefit. As a result, families with earnings above these thresholds would have experienced a greater reduction in income than the estimated 45% and 35% replacement rates associated with EI and long-term disability benefits, respectively.

Finally, the relatively small sample size limited the ability to conduct subgroup analyses examining the effects of socioeconomic status, insurance coverage, and other demographic factors. Larger studies are needed to better identify the populations at greatest risk of financial and psychosocial hardship during pediatric cancer treatment.

## Conclusion

The financial implications of cancer treatment can have devastating consequences to families during cancer treatment and lasting negative implications on families psychosocial and financial well-being. Current programs and infrastructure designed to support the financial needs of these families are falling short. Sustainable, equitable and consistent travel assistance programs for those at risk is required to all families regardless of location. More employment protections and access to longer employment insurance benefits, more flexible work schedules and financial support for those who are self-employed are needed to help families maintain an income to counter the increased financial burdens of cancer treatment while caring for their child. The current employment insurance benefits do not protect families of children with cancer from immense financial hardship.

## Supplemental Material

Supplemental material - A Longitudinal Observational Mixed Methods Cohort Study of the Psychosocial and Financial Costs of Cancer Care for Pediatric Families in the Maritimes, Canada Supplemental material for A Longitudinal Observational Mixed Methods Cohort Study of the Psychosocial and Financial Costs of Cancer Care for Pediatric Families in the Maritimes, Canada by Zara Nicole Forbrigger, Derek Bruce MacDonald, Tamara MacDonald in Cancer Control

## Data Availability

The datasets generated during and/or analyzed during the current study are available from the corresponding author on request.*
