# Supplementary material for: A Longitudinal Observational Mixed Methods Cohort Study of the Psychosocial and Financial Costs of Cancer Care for Pediatric Families in the Maritimes, Canada
Source: Cancer Control. 2026 Sep 11;33:10732748261468826. doi: 10.1177/10732748261468826 (PMC13569998; doi:10.1177/10732748261468826)
Supplement: Supplementary file 1 [file sj-pdf-1-ccx-10.1177_10732748261468826.pdf]

SUPPLEMENTAL TABLE 1 The number of estimated meals a participant would have based on the duration of their outpatient visit.

Number of hours	Meals
1-2 hours	0
3-5 hours	1
6-8 hours	2
9-11 hours	3

SUPPLEMENTAL TABLE 2 The rates used to calculate the mileage spent on coming to the IWK Health for cancer treatment by province and year.

Province	2016<br>¢/km	2017<br>¢/km	2018<br>¢/km	2019<br>¢/km	2020<br>¢/km
Nova Scotia	48.5	50.0	52.5	52.5	51.0
New Brunswick	48.5	50.5	53.5	53.5	50.5
Prince Edward Island	47.5	49.0	52.0	52.0	49.0

SUPPLEMENTAL TABLE 3 Parking fees for the IWK Health from 2016-2020

Time	Day price	Night price
1 hour	$3	$2
2 hours	$5	$4
3 hours	$7	$5
4 hours	$10	$5
5 hours	$12	$5
6-12 hours	$14	$5
1-7 days	$30	
8-14 days	$55	
15-21 days	$80	
22-28 days	$105	
29-35 days	$130	
36-42 days	$155	
43-49 days	$180	
50-56 days	$205	
57-64 days	$230	

SUPPLEMENTAL TABLE 4: The Confederation Bridge toll fees by year

Year	Cost
2016	$46.00
2017	$46.50
2018	$47.00
2019	$47.75
2020	$48.50
